# PVM/MA-shelled selol nanocapsules promote cell cycle arrest in A549 lung adenocarcinoma cells

**DOI:** 10.1186/s12951-014-0032-x

**Published:** 2014-08-23

**Authors:** Ludmilla Regina de Souza, Luis Alexandre Muehlmann, Mayara Simonelly Costa dos Santos, Rayane Ganassin, Rosana Simón-Vázquez, Graziella Anselmo Joanitti, Ewa Mosiniewicz-Szablewska, Piotr Suchocki, Paulo César Morais, África González-Fernández, Ricardo Bentes Azevedo, Sônia Nair Báo

**Affiliations:** 1 Institute of Biological Sciences, Molecular Biology Programme, University of Brasília, Brasília 70910-900, DF, Brazil; 2 Institute of Biological Sciences, University of Brasília, Brasília 70910-900, DF, Brazil; 3 Biomedical Research Center (CINBIO), Institute of Biomedical Research of Vigo, University of Vigo, Vigo 36310, Pontevedra, Spain; 4 Institute of Physics, Polish Academy of Sciences, Warsaw 02-668, Poland; 5 Department of Bioanalysis and Drugs Analysis, Warsaw Medical University, Warsaw 02-097, Poland; 6 Department of Pharmaceutical Chemistry, National Medicines Institute, Warsaw 00-725, Poland; 7 Institute of Physics, University of Brasília, Brasília 70910-900, DF, Brazil; 8 School of Automation, Huazhong University of Science and Technology, Wuhan 430074, Hubei, China

## Abstract

**Background:**

Selol is an oily mixture of selenitetriacylglycerides that was obtained as a semi-synthetic compound containing selenite. Selol is effective against cancerous cells and less toxic to normal cells compared with inorganic forms of selenite. However, Selol’s hydrophobicity hinders its administration *in vivo*. Therefore, the present study aimed to produce a formulation of Selol nanocapsules (SPN) and to test its effectiveness against pulmonary adenocarcinoma cells (A549).

**Results:**

Nanocapsules were produced through an interfacial nanoprecipitation method. The polymer shell was composed of poly(methyl vinyl ether-co-maleic anhydride) (PVM/MA) copolymer. The obtained nanocapsules were monodisperse and stable. Both free Selol (S) and SPN reduced the viability of A549 cells, whereas S induced a greater reduction in non-tumor cell viability than SPN. The suppressor effect of SPN was primarily associated to the G2/M arrest of the cell cycle, as was corroborated by the down-regulations of the CCNB1 and CDC25C genes. Apoptosis and necrosis were induced by Selol in a discrete percentage of A549 cells. SPN also increased the production of reactive oxygen species, leading to oxidative cellular damage and to the overexpression of the GPX1, CYP1A1, BAX and BCL2 genes.

**Conclusions:**

This study presents a stable formulation of PVM/MA-shelled Selol nanocapsules and provides the first demonstration that Selol promotes G2/M arrest in cancerous cells.

## Background

Low therapeutic efficacy and drug resistance are the most common problems related to the currently available chemotherapeutic agents used in tumoral clinical application. In the search for new chemotherapeutic drugs, several selenium (Se) compounds shown anticancer and anticarcinogenic activities [1],[2]. In particular, those containing Se at its 4+ oxidation state, namely selenite, present the highest antioxidant and anticancer activities [2]. However, Se(IV)-containing compounds generally present high systemic toxicity, limiting their clinical application. In this context, a selenite-containing compound named Selol, which was first obtained at Warsaw Medical University, Poland [3], has shown antitumor activity and low systemic toxicity [4],[5]. Selol is a mixture of different selenitetriacylglycerides and appears to act primarily through the induction of oxidative stress in cancer cells [5]. Interestingly, Selol was shown to sensitize leukemia cells to the cytotoxicity of vincristine and doxorubicin, insomuch that it was suggested that Selol could be used in combination with other drugs in chemotherapeutic protocols [4].

The potential synergism of Selol with classical anticancer drugs can be exploited to treat tumors, such as non-small cell lung cancer (NSCLC) [4]. NSCLC, whose most frequently observed histological subtype is adenocarcinoma, is particularly aggressive and the leading cause of cancer death worldwide [6],[7]. It is estimated that more than 75% of patients with NSCLC present locally advanced or metastatic disease, severely limiting the success of treatments [8],[9]. Platinum-based treatment, which is the most recommended first-line therapy, reaches response rates of only 20-40% and mean survivals between 7 and 12 months [8],[10],[11]. Multidrug protocols and a treatment break with non-platinum-based drugs after a fixed course of initial chemotherapy have been shown to prolong the survival of NSCLC patients [12],[13]. Thus, Selol may be a potential candidate for combined anti-NSCLC strategies [14].

Despite the therapeutic potential presented by Selol, its hydrophobicity is a major obstacle to its biological application. For instance, high hydrophobicity often hinders intravenous (iv) administration and may thus confer an undesirable pharmacokinetic profile [15]. This problem can be circumvented through the nanoencapsulation of Selol in an aqueous vehicle to form a nanocapsule-based drug delivery system. The nanoencapsulation of Selol with a polymer presenting highly reactive chemical groups allowing surface modification could bring up new possibilities for delivering this anticancer agent. In this context, the copolymer poly(methyl vinyl ether-co-maleic anhydride) (PVM/MA) has been reported to be a biocompatible and biodegradable material useful for preparing drug delivery systems [16]. Additionally, the copolymer PVM/MA presents a surfactant effect and anhydride groups, which readily react with a series of molecules. On that ground, we report the development and the first *in vitro* efficacy tests of a PVM/MA-shelled Selol nanocapsule formulation intended for the treatment of lung adenocarcinomas.

## Results and discussion

### Formulation screening

The interfacial precipitation method of preformed polymer through solvent displacement yields nanosized Selol capsules only within a certain range of solute and solvent concentrations. Thus, to identify the best formulation parameters, different concentrations of each component were tested.

**Figure 1:**
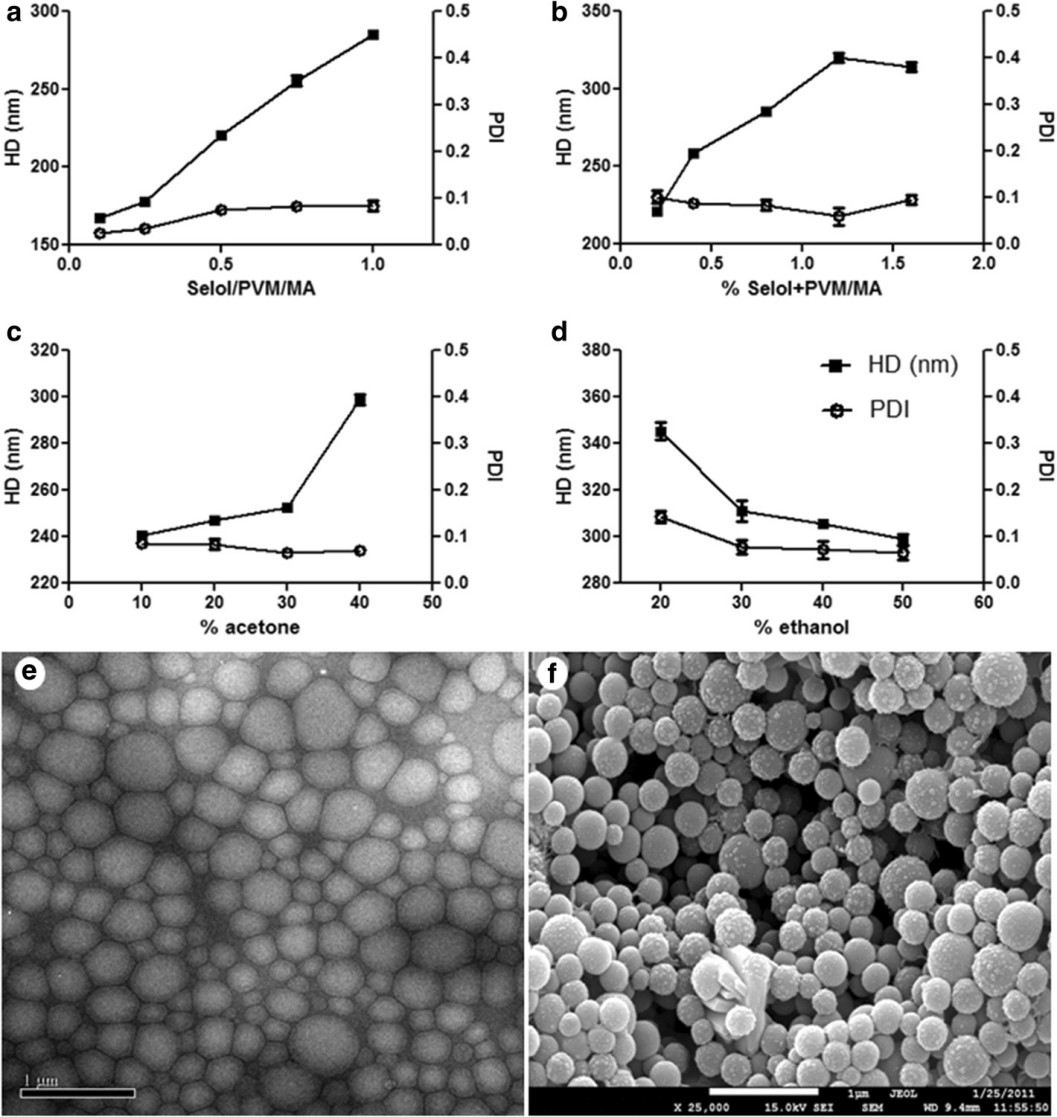
**Characterization of Selol nancapsules.** Values of the hydrodynamic diameters (HD) (in squares) and polydispersity indexes (PDI) (in diamond) of PVM/MA-shelled Selol nanocapsules as a function of the Selol-to-PVM/MA ratio **(a)**, concentration of Selol plus PVM/MA **(b)**, acetone **(c)**, and ethanol **(d)**. Morphology of Selol-PVM/MA nanocapsules (SPN) observed by TEM **(e)** and SEM **(f)**. Magnification: 25.000 × .

First, different Selol-to-PVM/MA ratios were tested (0.1, 0.2, 0.5, 0.7, 1.0, 1.5, 2.0, and 4.0, w:w), and the concentrations of acetone, ethanol and water were fixed to 20, 40 and 40% (v:v), respectively. As shown in Figure 1(a), the increase in this ratio led to a directly proportional increase in the hydrodynamic diameter (HD) values of the nanocapsules. Using Selol-to-PVM/MA ratios from 0.1 to 1.0, monodisperse nanocapsule populations were obtained, and the polydispersity index (PDI) values remained below 0.1. Formulations prepared with Selol-to-PVM/MA ratios higher than 1.0 showed visible decantation minutes after preparation and were not used for dynamic light scattering analysis. Therefore, this parameter was set to 1.0 for the next steps because this was the highest value that allowed stable nanocapsules to be obtained.

Next, different concentrations of Selol plus PVM/MA were tested. The concentrations of acetone, ethanol and water were set to 20, 40 and 40% (v:v), respectively. As expected, smaller nanocapsules were obtained at the lowest concentrations of Selol plus PVM/MA (Figure 1(b)). The PDI was not significantly affected by this variable, remaining close to 0.1. The concentration of Selol plus PVM/MA was set to 0.8% (w:v) in further experiments because it provided good colloidal characteristics in addition to a good yield of nanocapsules.

Then, different concentrations of acetone and ethanol were tested in the process of encapsulation with 0.8% Selol plus PVM/MA and a ratio of 1.0 Selol-to-PVM/MA. Different volumes of acetone were used for dissolving a fixed amount of Selol and PVM/MA, and the final volume reached 100% with ethanol:water (1:1, v:v). As shown in Figure 1(c), a major change in the nanocapsule HD was observed with 40% acetone, but the PDI remained below 0.1. When the concentration of acetone was set to 20% and varying volumes of ethanol were added, it was observed that the HD of the nanocapsules decreased with higher concentrations of ethanol (Figure 1(d)). The highest HD and PDI values were obtained with 20% ethanol.

Given the results described above, the protocol of Selol nanoencapsulation was established as follows: 1) 100 mg of PVM/MA and 100 mg of Selol were dissolved in 5 mL of acetone; 2) 10 mL of ethanol and 10 mL of water were added; and 3) the purification steps were then performed.

The method of nanoprecipitation by solvent displacement yielded monodisperse nanocapsules at almost all of the conditions tested and also allowed modulation of the nanocapsule diameter. Noteworthy, by varying the concentrations of acetone and ethanol, nanocapsules of different HDs were obtained, likely due to differences in solvent diffusion, as previously suggested [17]. Even for a concentration of nanocapsule components (Selol plus PVM/MA) near the upper critical limit of 2%, as noted by Aubry et al. (2009) [18] for this method, stable and monodisperse nanocapsules were obtained. As expected, at higher concentrations of Selol, larger capsules were obtained, which can be attributed to the nucleation-and-growth phenomenon [19],[20].

### Characterization of Selol nanocapsules

The Selol nanocapsules (SPN) formulation presented a single population of nanocapsules with an HD of 344.4 ± 4.8 nm, a PDI of 0.061 ± 0.005 and a zeta potential (ζ po tential) of −29.3 mV ± 1.5. The transmission electron microscope (TEM) image revealed a population of nanocapsules with an average diameter of 207.9 ± 80.9 nm (Figure 1(e)). These nanometric structures presented a spherical shape and slightly rough surface, as observed with a scanning electron microscope (SEM) (Figure 1(f)). A spherical equilibrium shape is expected with this method due to the three-dimensional primordial droplet nuclei growth conferred by the interfacial tension between the droplets and the dispersant [20],[21]. Furthermore, according to the TEM images, the Selol nanocapsules appear to be deformable. Both the deformability and the spherical shape of the nanocapsules are interesting for their administration through parenteral routes [22].

**Figure 2:**
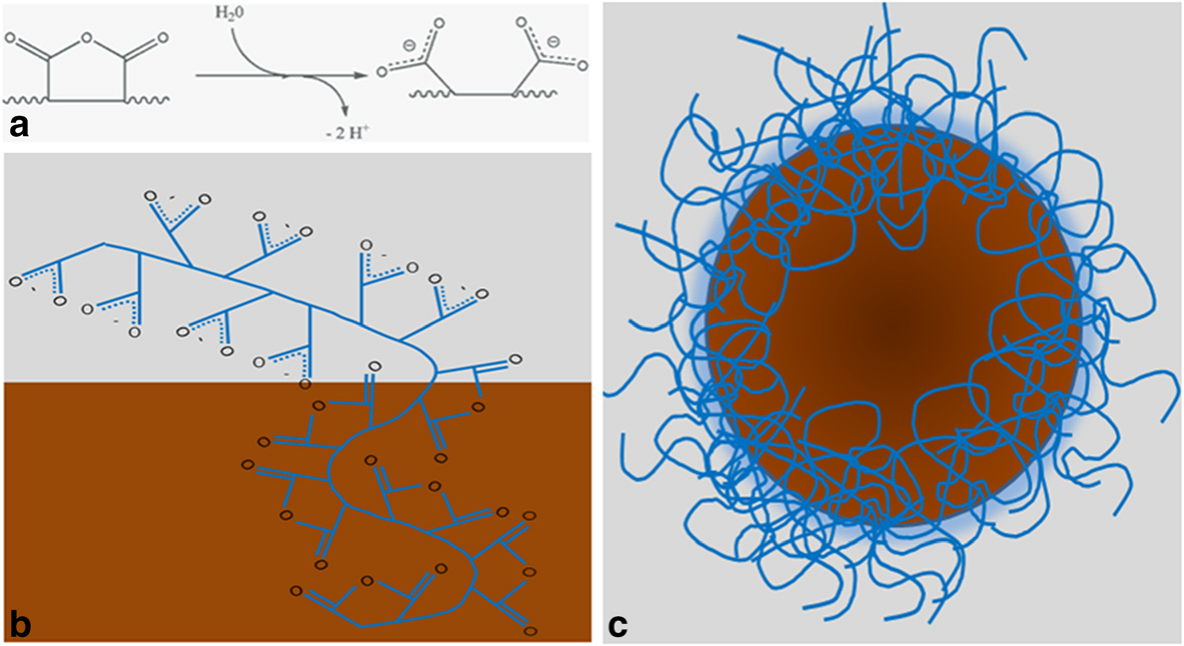
**Schematic representation of PVM/MA-shelled Selol nanocapsules.** Hydrolysis of an anhydride group yields two carboxylate groups at neutral pH in a PVM/MA strand **(a)**. Partial hydrolysis of the PVM/MA polymer strand exposed to water but not in PVM/MA closer to the oily core **(b)**. The carboxylate-containing parts of PVM/MA are hydrophilic and comprise the nanocapsule shell surface, whereas the anhydride-containing parts are more hydrophobic, closely covering the Selol core **(c)**.

The dosage of the purified Selol nanocapsules showed that the concentration of Selol measured in the nanocapsule was approximately 100% of the initial amount of Selol used in the process. Supported by the low PDI of the Selol composition, this result confirms that Selol was efficiently encapsulated into the PVM/MA shell. However, it was necessary to evaluate the copolymer’s capacity for interfacial stabilization after prolonged periods of storage and thermal stress conditions. As observed in Table 1, the SPN was stable at room temperature (RT) for at least 60 days. Under these conditions, there was no statistically significant change in the HD over the evaluated period of time. When stored at 4°C, the SPN average HD increased by approximately 40 nm on the first day and then remained stable. At both RT and 4°C, the PDI remained well below 0.1, showing that the nanocapsules remained monodisperse. At −20°C, significant variations were observed in both the HD and PDI (>0.3), indicating that this condition is not adequate for storage. After freezing/thawing cycles, no significant changes in both the HD and PDI (p > 0.05) were found up to the eighth cycle. Indeed, PVM/MA presented good capacity for interfacial stabilization in SPN because this system did not present any important changes in its characteristics, neither after 60 days of storage (at RT or 4°C) nor after being subjected to the thermal stress of eight cycles of freezing/thawing. The ζ potential of the nanocapsules was generally between −25 and −30 mV at all of the conditions tested, which is due to the presence of carboxylate groups on their outer surface. A hypothetical scheme of the PVM/MA-shelled Selol nanocapsules is shown in Figure 2*.* Over the course of encapsulation, the PVM/MA copolymer, which is poorly soluble in water, becomes amphipathic due to the hydrolysis of some of its anhydride groups exposed to water. The formed polymer shell presents a hydrophilic water-exposed surface, facilitating the stabilization of nanocapsules.

**Table 1:** Thermodynamic stability and dispersibility studies of Selol-PVM/MA nanocapsules

**Formulation**	**Hydrodynamic diameter ± S.D. (nm)**	**Polydispersity index**	**Zeta potential (mV)**
Room temperature			
Day 0	344.4 ± 4.8^a^	0.061	−29.3
Day 15	349.7 ± 8.2^a^	0.049	−29.7
Day 30	338.9 ± 6.4^a^	0.046	−29.2
Day 45	344.3 ± 2.0^a^	0.034	−26.5
Day 60	336.6 ± 4.6^a^	0.043	−29.4
4°C			
Day 0	344.4 ± 4.8^a^	0.061	−29.3
Day 1	390.2 ± 1.0^b^	0.054	−29.3
Day 15	391.3 ± 7.5^b^	0.044	−25.1
Day 30	373.9 ± 15.0^b^	0.046	−27.8
Day 45	381.6 ± 8.9^b^	0.031	−30.1
Day 60	380.6 ± 7.1^b^	0.039	−27.3
−20°C			
Day 0	344.4 ± 4.8^a^	0.061	−29.3
Day 1	414.2 ± 7.7^c^	0.074	−29.2
Day 15	408.2 ± 10.7^c^	0.113	−27.0
Day 30	471.0 ± 1.3^d^	0.195	−27.4
Day 45	615.2 ± 13.3^e^	0.278	−29.8
Day 60	566.3 ± 14.8^f^	0.196	−26.5
Freezing (−20°C) and thawing cycle			
No cycle	344.4 ± 4.8	0.061	−29.3
I	414.2 ± 7.74	0.074	−29.2
II	414.1 ± 10.0	0.066	−28.9
III	406.7 ± 4.5	0.035	−28.9
IV	410.2 ± 4.7	0.045	−28.1
V	416.5 ± 4.3	0.064	−22.9
VI	405.5 ± 6.0	0.046	−28.5
VII	425.8 ± 0.9	0.079	−28.8
VIII	467.3 ± 9.9	0.143	−27.0
IX	486.5 ± 17.1^*^	0.106	−28.8
X	495.1 ± 1.0^*^	0.177	−30.8
XI	509.3 ± 6.6^*^	0.221	−28.9
XII	566.2 ± 3.5^*^	0.364	−28.7
XIII	865.1 ± 50.4^*^	0.581	−27.7
XIV	989.1 ± 26.6^*^	0.433	−26.9
XV	1027.5 ± 60.7^*^	0.393	−28.8

All values were expressed as the means ± standard error of the mean. Different letters indicate significant differences between SPN treatment for one day at RT compared with a given time and/or storage condition (p < 0.05). *Significantly different compared with the first cycle of freezing (−20°C) and thawing (p < 0.05).

### Selol nanocapsules affect cell viability in a concentration- and time-dependent manner

**Figure 3:**
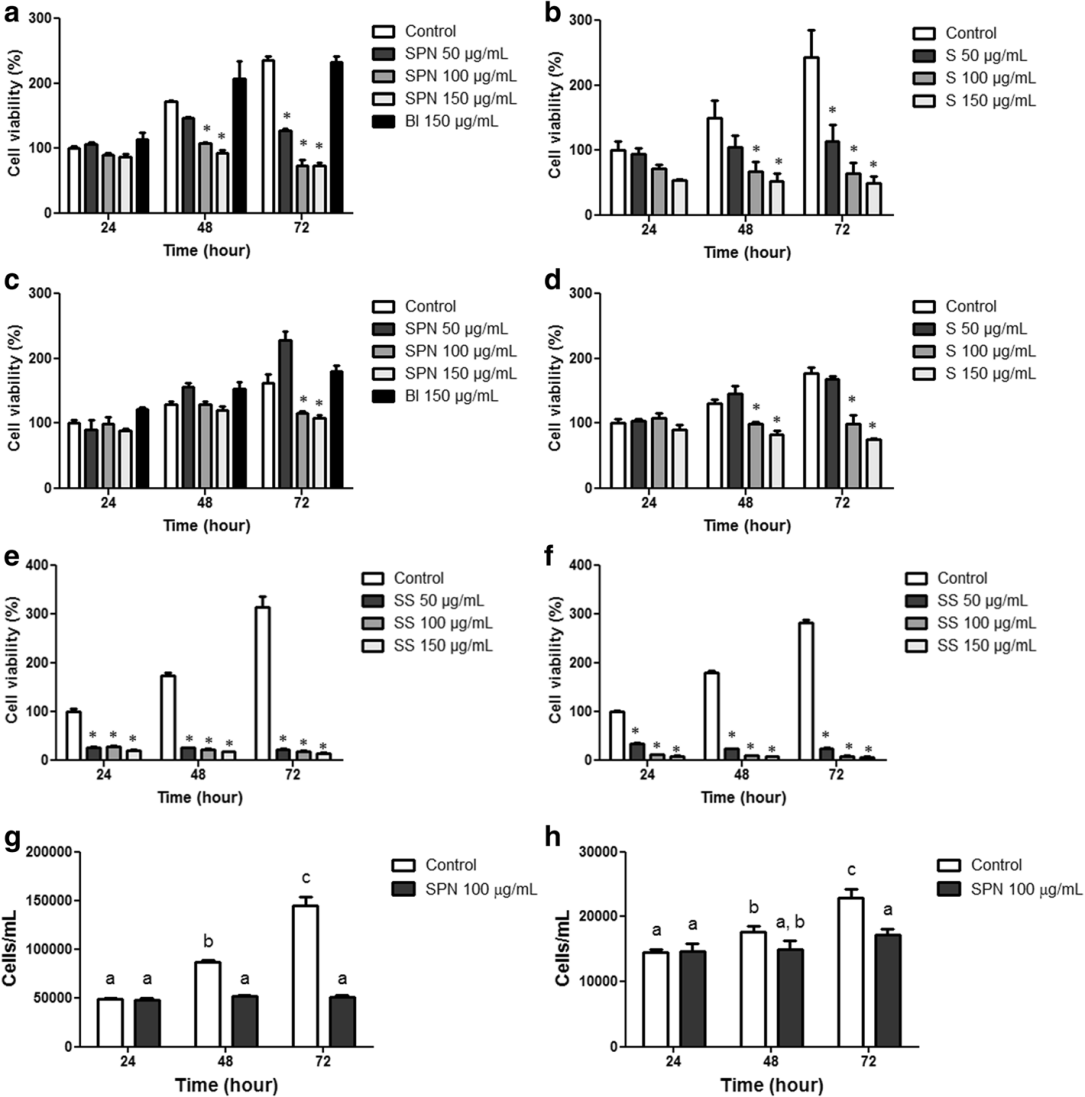
**Viability of A549 (a, b, e) and human connective tissue (c, d, f) cells after exposure to Selol-PVM/MA nanocapsules (SPN) (a, c), free Selol (S) (b, d), blank polymeric nanoparticles without Selol (Bl) (a, c) or sodium selenite (SS) (e, f) at 50, 100 and 150 μg/mL Se for 24 h, 48 h and 72 h.** All of the values were normalized according to the control group at 24 h (100%). The time-dependent proliferation of control cells was significantly different. *Statistically significant compared with the control group at the corresponding treatment period (p < 0.05). Influence of SPN at a concentration of 100 μg Se/mL on the proliferation of A549 **(g)** and human connective tissue **(h)** cells evaluated after different treatment times. Pairs of means in a same graph identified with different letters are significantly different (p < 0.05).

The MTT assay showed that nanoparticles of PVM/MA without Selol (Bl) did not significantly affect the viability of the studied cells (Figure 3). Significant reductions in the viability of human lung adenocarcinoma (A549) cells were observed after exposure to SPN or free Selol (S) at concentrations of 50 μg/mL for 72 h and 100 and 150 μg/mL for 48 and 72 h (p < 0.05). In contrast, tissue connective normal cells were shown to be less sensitive to Selol compared with tumor cells. Free Selol treatment reduced the viability of normal cells after 48 h, whereas SPN reduced their viability only after 72 h of incubation. Sodium selenite (SS) was highly toxic for both cell types at all concentrations and times tested. The high toxicity of SS toward normal cells shows that selenite is an effective anticancer agent but is not safe for clinical use in its inorganic form. Therefore, some researchers have aimed to find organic selenium compounds with higher therapeutic indexes [23],[24]. Selol, an organic selenite compound, significantly reduced the viability of lung adenocarcinoma A549 cells but was far less toxic on normal cells than sodium selenite. This finding reinforces the previously shown evidence that Se (4+) organified as Selol has a significantly lower potential to exert deleterious effects on non-target tissues than its inorganic form [4],[25],[15]. Noteworthy, the encapsulation of Selol did not significantly affect its activity against A549 cells and reduced its toxicity toward normal cells. These results encourage further tests of Selol activity in *in vivo* models of pulmonary cancer. Moreover, some characteristics of the investigated nanocapsules point to their potential to act as good drug delivery systems. First, their hydrodynamic diameters can be tuned to values that allow for the passive targeting of tumors via an enhanced permeation and retention effect (EPR) because the tumor microvasculature usually presents pores with diameters of 100 to 780 nm [26]. Second, targeting molecules can be conjugated to the surface of nanocapsules to increase their affinity to cancerous cells. This procedure can be easily performed because PVM/MA has anhydride groups, which can easily react with the hydroxyl or primary amine groups present in most of the available targeting molecules [16].

**Figure 4:**
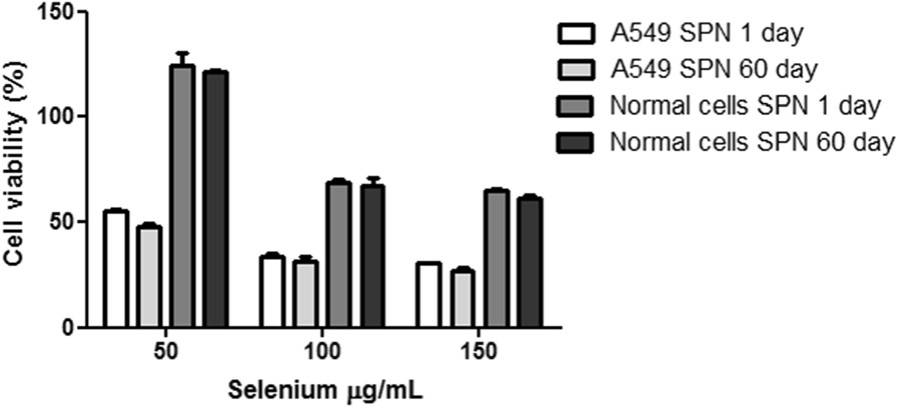
**Viability of A549 and human connective tissue cells treated with Selol-PVM/MA nanocapsules (SPN) after 1 and 60 days of storage at 4°C.** The cells were treated with 50, 100 and 150 μg/mL Se for 72 h, and the data are expressed as the means ± standard error of the mean of the percentages of viable cells. No statistically significant difference was found between the results for SPN after 1 day and SPN for 60 days (p > 0.05).

Subsequent experiments with SS, S and SPN were performed with a concentration of 100 μg/mL Se because this was the lowest concentration in S and SPN treatments that reduced cell viability. Moreover, SPN was always used within 60 days after preparation because its biological activity did not significantly change during storage at 4°C for this period of time (p > 0.05) (Figure 4).

### SPN reduces cell proliferation and promotes cell cycle arrest

As expected, both untreated normal and A549 cells proliferate in culture, as evidenced by the time-dependent increase in the number of cells (p < 0.001 for both 24 vs. 48 h and 48 vs. 72 h for A549; p < 0.01 for 24 vs. 48 h, and p < 0.05 for 48 vs. 72 h for normal cells) (Figure 3 (g and h)). SPN treatment inhibited the proliferation of both cell types, but the intensity of this effect was cell line-dependent. The number of both normal and tumor cells in the SPN-treated groups did not vary over the treatment time (p > 0.05). Reductions of 40.4 ± 2.7% and 64.7 ± 1.7% in the number of SPN-treated A549 cells were evidenced at 48 h (p < 0.001) and 72 h (p < 0.001), respectively, relative to the control A549 cells. However, normal cells were affected to a lesser extent because a significant reduction in their number was observed only at 72 h (24.8 ± 6.7%, p < 0.01) compared with the control normal cells.

**Figure 5:**
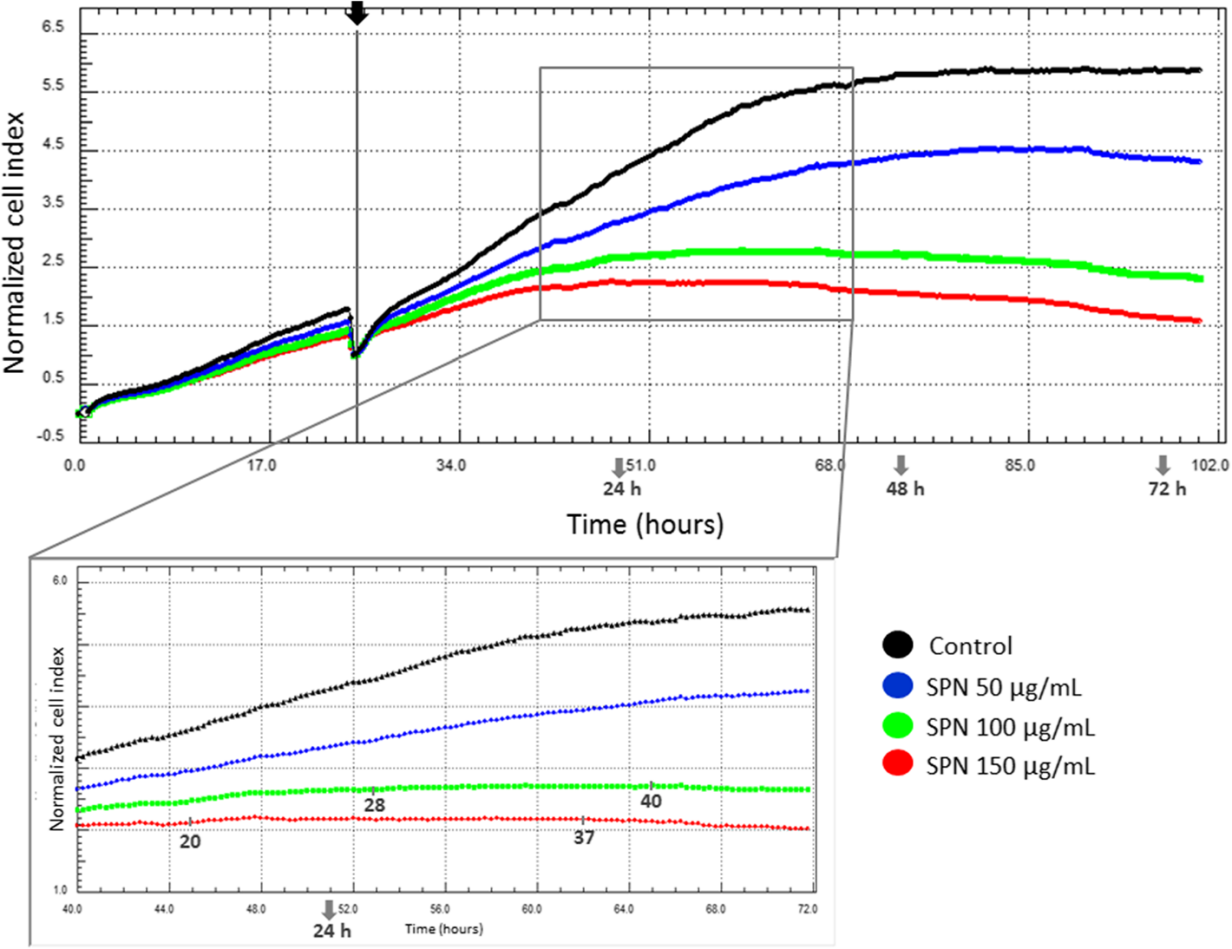
**Real-time monitoring of cell indexes of A549 cells untreated (control) and treated with Selol-PVM/MA nanocapsules (SPN) at Se concentrations of 50, 100 and 150 μg/mL for 100 h.** All of the treatments started 24 h after cell plating, as indicated by the vertical grey continuous line on the top panel. The bottom panel shows more detailed dynamic monitoring in the range of 15 h to 47 h after the treatment, and the time intervals with constant cell indexes are indicated on the bottom panel (values should be related to the treatment and not to the time after cell plating).

The real-time cell index monitoring also showed changes in this parameter after the first hours of SPN treatment on A549 cells, which became more significant for higher concentrations and longer times of incubation (Figure 5). Cell growth was detected up to 20 h, 28 h and 56 h in the presence of SPN at 150 μg/mL, 100 μg/mL and 50 μg/mL concentrations, respectively. Over these times, the cell indexes remained constant and subsequently decreased. Similar to previous results, the cell index in the treatment with 100 μg/mL SPN was almost invariant at 24, 48, and 72 h.

**Figure 6:**
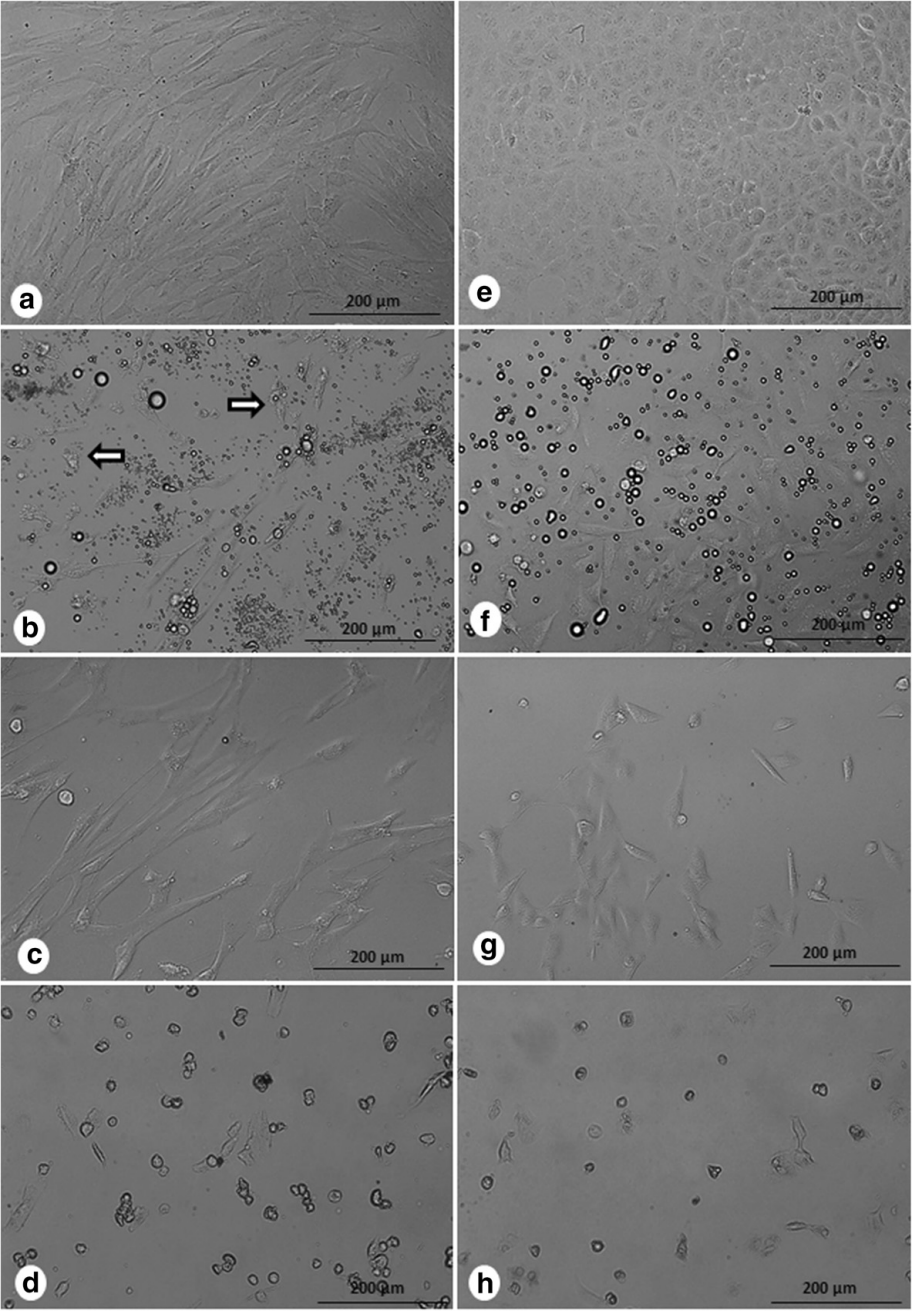
**Morphology of human cells from connective tissue (a, b, c, d) and A549 cells (e, f, g, h).** The cells were untreated **(a, e)**, exposed to free Selol (S) **(b, f)** or to Selol-PVM/MA nanocapsules (SPN) **(c, g)** for 72 h. Morphologic changes due to sodium selenite (SS) treatment for 24 h **(d, h)**. Decrease in cell confluence by S and SPN treatments. Normal morphology of A549 **(f, g)** and SPN-treated connective tissue cells. Cytoplasmic retraction (arrows) on human cells from connective tissue after treatment with S **(b)**. The bright, black-bordered spherical structures are free Selol microdroplets **(b, f)**. Magnification: 20x.

**Figure 7:**
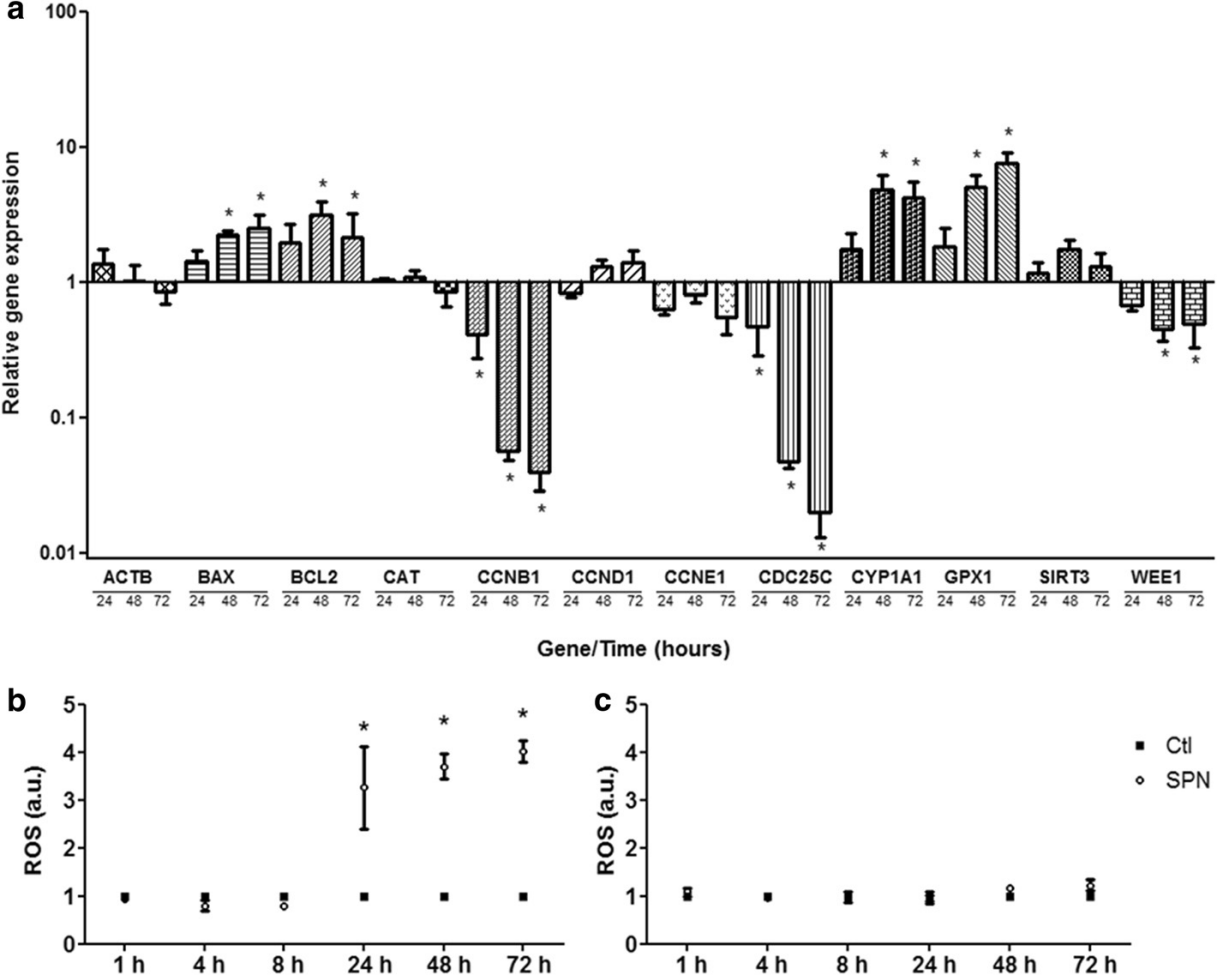
**Relative gene expression of A549 cells treated with Selol-PVM/MA nanocapsules (SPN) at a concentration of 100 μg/mL for 24 h, 48 h and 72 h (a).** The following genes were down-regulated: CCNB1, CDC25C and WEE1. The following genes were up-regulated: BCL2, BAX, GPX1 and CYP1A1. *FC > 2.0. Production of reactive oxygen species (ROS) in A549 **(b)** and HL60 **(c)** cells treated with SPN at a concentration of 100 μg/mL (SPN). SPN-treated samples were normalized according to the control samples (Ctl). The bars represent the means ± standard error. *p < 0.001.

These results were corroborated by light microscopy images, which showed a more intense reduction in the confluence of SPN-treated A549 cells compared with SPN-treated normal cells (Figure 6). A549 cells treated with SPN presented a significantly higher proportion of cells arrested at the G2/M phase compared with that obtained for control cells (Table 2). These results are in good agreement with the decreases in the gene expression levels of CCNB1 (cyclin B1), CDC25C and WEE1 (FC (fold change) > 2.0) and unchanged expression levels of the gene transcripts of CCND1 (cyclin D1) and CCNE1 (cyclin E1) (FC < 2.0) (Figure 7(a)), indicating that Selol acts on the G2/M arrest of the cell cycle and has no activity on the G1 and S checkpoints. In response to SPN treatment, the expression levels of CCNB1 and CDC25C were reduced by half at 24 h and were markedly down-regulated after 48 h (approximately 17-fold for CCNB1 and 21-fold for CDC25C) and 72 h (approximately 25-fold for CCNB1 and 50-fold for CDC25C) compared with the control cells. The expression of WEE1 was reduced only to half at 48 h and 72 h.

**Table 2:** Effect of Selol-PVM/MA nanocapsule (SPN) treatment on the cell cycle distribution of A549 cells

**A549**	**G0/G1 (%)**	**S (%)**	**G2/M (%)**
**Control 48 h**	69.6 ± 2.9	18.4 ± 2.3	12.2 ± 1.3
**SPN 48 h**	56.6 ± 3.8	26.5 ± 0.9	16.9 ± 3.7*
**Control 72 h**	70.7 ± 3.9	18.8 ± 2.9	10.6 ± 1.7
**SPN 72 h**	56.2 ± 1.3	25.0 ± 1.9	18.7 ± 2.7*

*p < 0.05 compared with the control. The data were obtained from three independent experiments and are presented as the means ± standard error of the mean.

In relation to normal cells, A549 cells were more susceptible to Selol, both free and encapsulated, which may be partially due to enhanced endocytic activity [27]. Another possible explanation that is supported by the results of the present study is that tumor cells may be more sensitive to Selol due to their higher proliferation rates because Selol appears to primarily act as an inhibitor of cell proliferation. SPN significantly increased the percentage of A549 cells arrested in the G2/M phase of cell cycle and consequently reduced the number of living cells. In the G2 phase, the wee1 protein inactivates the mitosis promoting factor (cyclin B1/CDK1), and cdc25C is a positive regulator of this complex. As shown in previous studies, G2/M arrest may require activation of wee1 in addition to inactivation of cdc25C [28],[29].

Additionally, the decrease in cell proliferation induced by Selol was not associated with a decreased energy metabolism coordinated by the main mitochondrial deacetylase sirtuin 3 [30], as observed by the unchanged transcript levels of the SIRT3 gene (FC < 2.0) (Figure 7(a)). Moreover, subtle changes in the expression of the β-actin gene (ACTB) in SPN-treated cells compared with untreated cells showed that this gene is not suitable as an endogenous control for SPN treatment (Figure 7(a)), likely due to association of the β-actin protein with cytoskeletal components and consequent cell division events [31].

### Morphological alterations

Phase contrast microscopy revealed that neither SPN nor free Selol induced morphological changes in A549 cells. SPN treatment did not induce any morphological alterations in normal cells because most of them were shown to be spindle-shaped with cytoplasmic projections, as expected. In contrast, free Selol induced visible cytoplasmic retraction (arrows) in normal cells (Figure 6). Comparatively, pronounced morphological changes were induced by SS in both normal and A549 cells.

**Figure 8:**
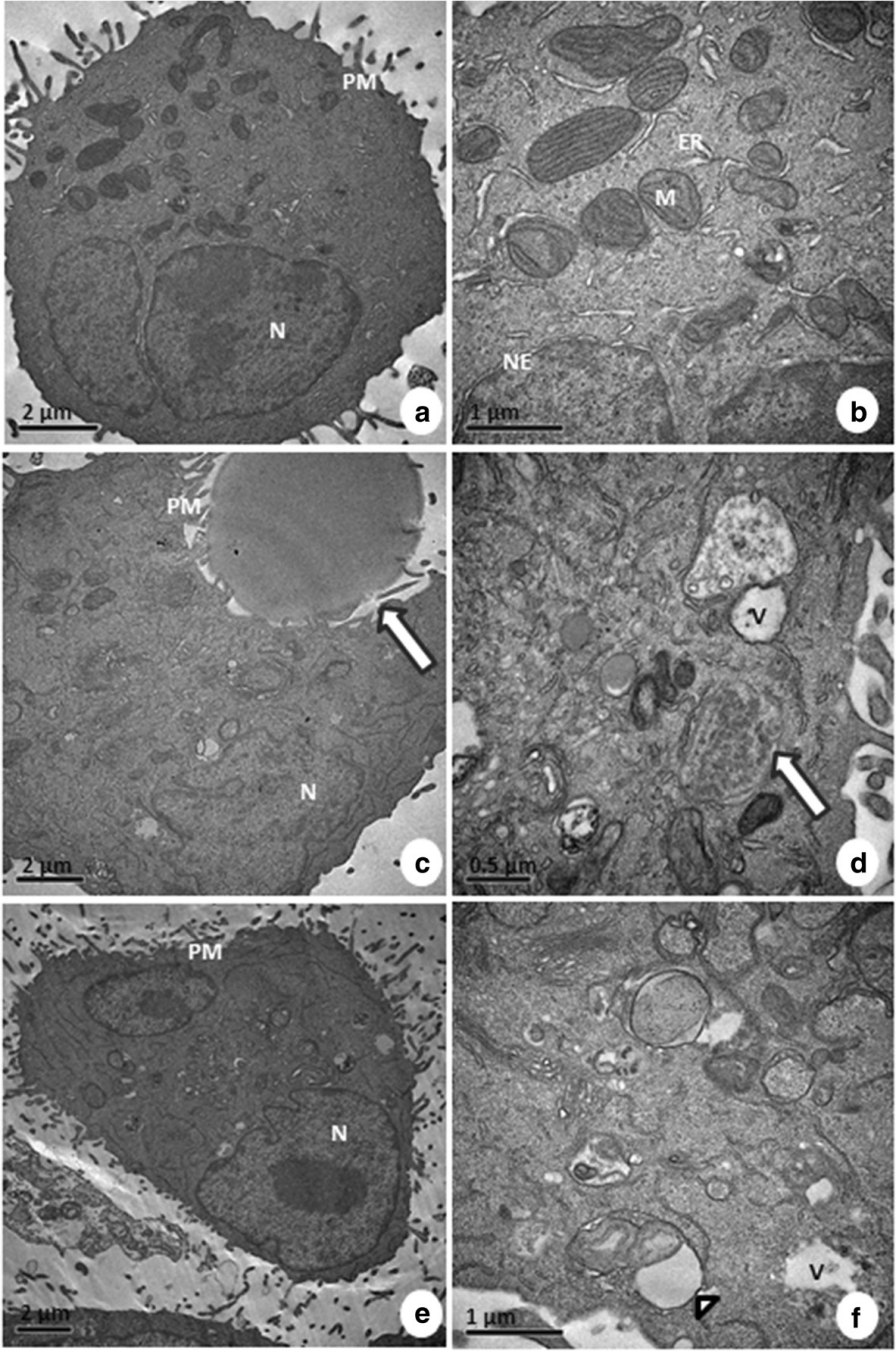
**Ultrastructural morphology of A549 cells: control (a, b), treated with free Selol (S) (c, d) and treated with Selol-PVM/MA nanocapsules (SPN) (e, f).** Note the integrity of the mitochondria (M), endoplasmic reticulum (ER), plasma membrane (PM), nucleus (N), nuclear envelope (NE), and cytosol of the control cells. An endocytosed Selol droplet is shown with an arrow in **c**. Endosomes, as indicated by an arrow in **d**, were usually identified with the free Selol treatment. Cytoplasm characteristic of necrotic cells can be observed in **e** (arrow). Intense formation of vacuoles (V) containing cellular organelles and changes in the morphology of mitochondria (arrowhead) can be evidenced in **d** and **f**.

**Figure 9:**
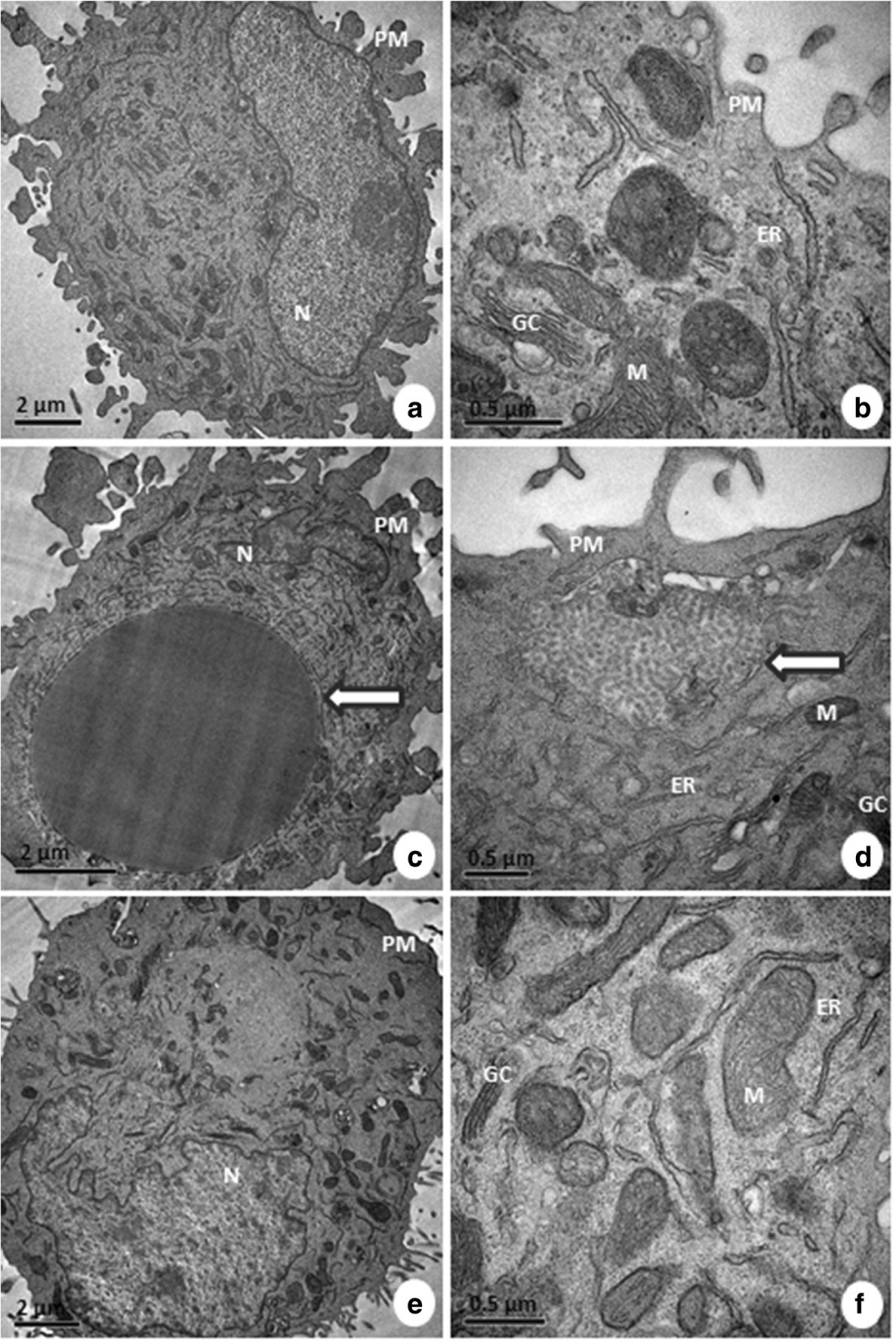
**Ultrastructural morphology of human connective tissue cells: control (a, b), treated with free Selol (S) (c, d) or treated with Selol-PVM/MA nanocapsules (SPN) (e, f).** Note the integrity of the control cells, as expected. In **c**, a Selol vesicle being taken up by a cell exposed to free Selol is shown by an arrow. In **d**, the arrow points to an endosome. In **e** and **f**, the ultrastructural morphology is unchanged by SPN treatment. N, M, ER, GC, PM, and V: nucleus, mitochondria, endoplasmic reticulum, Golgi complex, plasma membrane and vesicle, respectively.

Some ultrastructural signs of cell death were observed in A549 cells after treatment with Selol. Mitochondrial changes and intense vacuolization were apparent (Figure 8). Additionally, cytoplasm swelling suggestive of necrosis was present. These morphological changes suggest that apoptosis and necrosis are induced by treatment with Selol. Most of the normal cells did not present any morphological changes after SPN treatment (Figure 9). However, the cells exposed to free Selol presented large endosomes containing Selol, whereas the SPN-treated cells did not present visible Selol particles. Endocytosis of free Selol droplets in both cell lines caused the compression of adjacent organelles. In addition, an unambiguous identification of Selol nanoparticles was not possible due to similarities with cellular lipids.

### Cell death signaling

**Figure 10:**
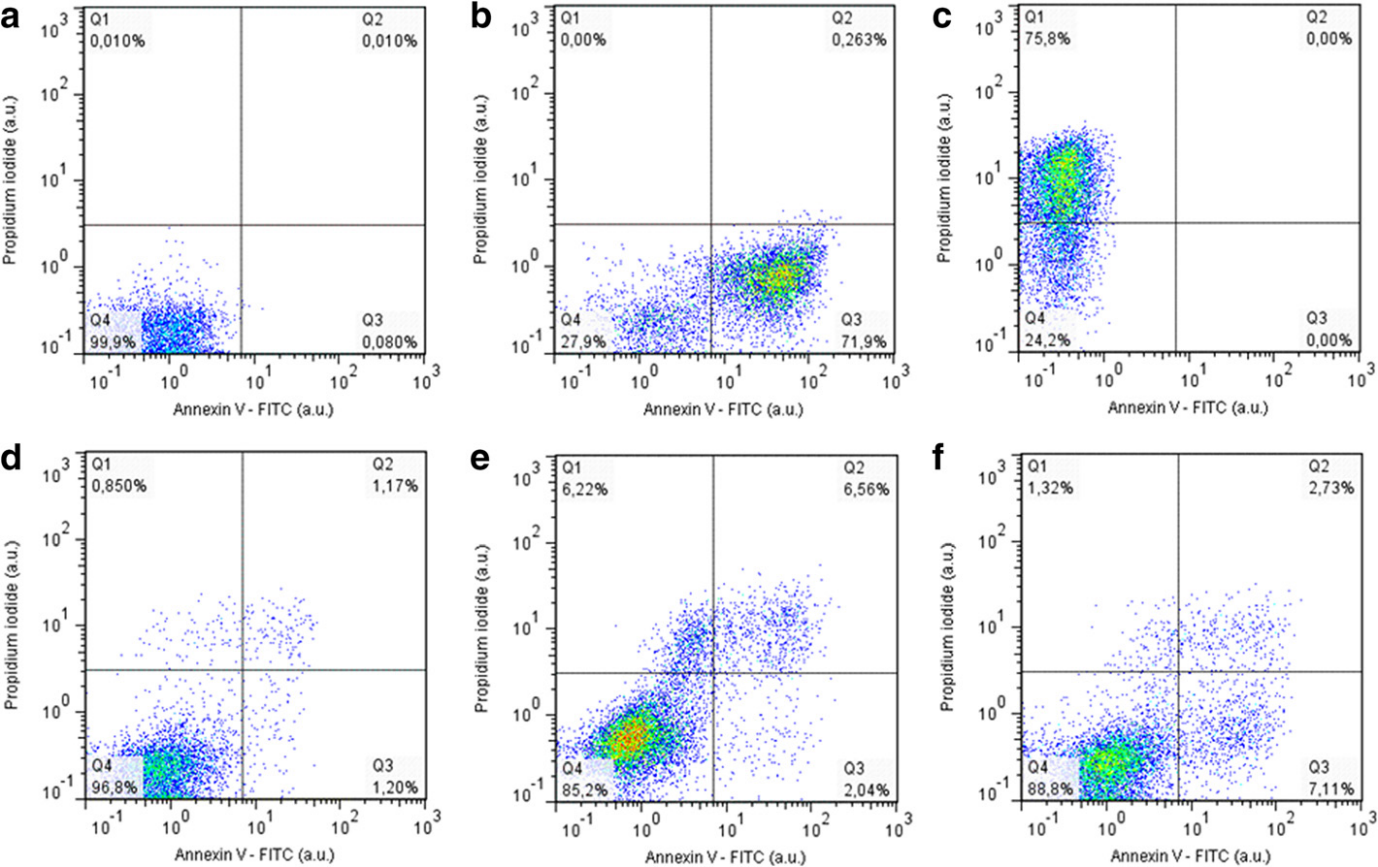
**Influence of free Selol (S) or Selol-PVM/MA nanocapsules (SPN) on A549 cells.** Flow cytometry study of cells stained with propidium iodide (PI) and/or annexin V-FITC. The positive cells are shown as percentages in each quadrant: PI^+^ (Q1), Annexin V^+^ (Q3), double-negative PI^−^ Annexin V^−^ (Q4), and double-positive cells PI^+^ Annexin V^+^ (Q2). Negative control **(a)**. Annexin V^+^ control **(b)**. PI^+^ control **(c)**. Untreated cells **(d)**. Similar membrane integrity was observed after S **(e)** and SPN **(f)** treatments.

S and SPN induced the exposure of phosphatidylserine on the outer leaflet of the plasma membrane and impairment of plasma membrane permeability only in a discrete percentage of A549 cells. After 72 h of exposure, only 11.2 ± 0.6% (p < 0.01) and 12.9 ± 2.3% (p < 0.001) of A549 cells were propidium iodide (PI)- and/or annexin V-positives after S and SPN treatments, respectively. In control A549 cells, this percentage was 2.9 ± 0.5% (Figure 10).

**Figure 11:**
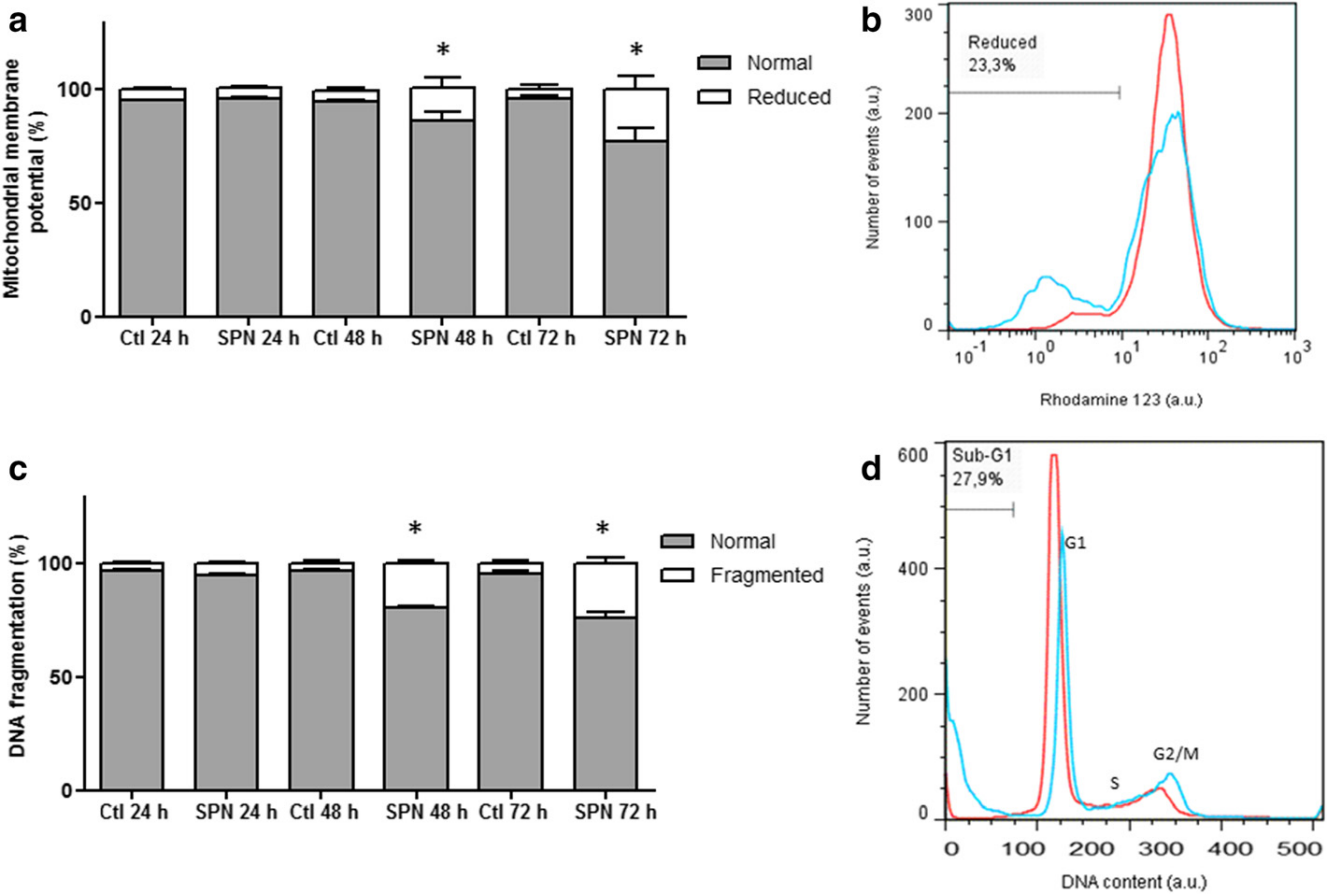
**Mitochondrial membrane potential and by DNA fragmentation of A549 cells treated with selol-PVM/MA nanocapsules (SPN) and untreated cell (control cells, Ctl) at different times.** *p < 0.05 for Ctl 48 h vs. SPN 48 h; p < 0.001 for Ctl 72 h vs. SPN 72 h **(a)**. Representative histogram showing a sample of control cells (red line) and a sample of SPN-treated cells (blue line) after 72 h **(b)**. DNA fragmentation in SPN-treated or untreated A549 cells at different times. *p < 0.001 for Ctl 48 h vs. SPN 48 h; p < 0.001 for Ctl 72 h vs. SPN 72 h **(c)**. Representative histogram showing sub-G1 (DNA fragmented), G1, S and G2/M populations for Ctl (red line) and SPN-treated cells (blue line) after 72 h **(d)**.

SPN treatment caused an increase in the percentage of A549 cells presenting reduced mitochondrial membrane potential (*∆Ψm*) compared with control cells at 48 h (14.5 ± 4.2%, p < 0.05) and 72 h (22.8 ± 5.4%, p < 0.001) (Figure 11(a)). Figure 11(b) also shows the *∆Ψm* of control and SPN-treated A549 cells at 72 h. These findings are compatible with the mitochondrial damages evidenced by ultrastructure analysis.

DNA fragmentation increased after 48 h (19.8 ± 1.1% vs. 3.6 ± 1.0% for control, p < 0.001) and 72 h (24.0 ± 2.5% vs. 4.6 ± 1.0% for control, p < 0.001) of SPN treatment (Figure 11(c)). As shown in Figure 11c, this treatment resulted in an increase in the sub-G1 cell population, a clear reduction in the percentage of cells in the G1 phase and an increase in the percentage of cells in the G2/M phase (Figure 11(d)).

The levels of transcripts related to apoptosis, namely BAX (BCL2-associated X protein) and BCL-2 (B-cell lymphoma 2), increased after 48 h (2-fold and 3-fold for BAX and BCL-2, respectively) and 72 h (2-fold for both BAX and BCL-2) (Figure 7(a)). These results suggest that Selol triggers damages that are able to activate the apoptosis mechanisms. However, the BAX/BCL2 ratio, which is recognized as an initiator of the caspases activation pathway, was slightly increased only at 72 h. The results presented suggest that the mechanism of action of Selol in A549 cells is not crucially dependent on the direct induction of apoptosis or necrosis. Suchocki et al. (2007) [4] demonstrated mitochondrial changes and DNA fragmentation in a leukemia cell line, and Estevanato et al. (2012) [32] and Wilczynska et al. (2011) [25] observed the translocation of phosphatidylserine in breast and cervix cancer cell lines. In the present study, the exposition of phosphatidylserine, changes in the mitochondrial membrane potential, DNA fragmentation, and/or alterations in the plasma membrane permeability were observed in low percentages of A549 cells after treatment with free or encapsulated Selol. These findings, however, do not exclude the possibility that Selol may induce extensive A549 cell death at the highest concentrations and/or after prolonged treatment times. Prolonged cell cycle arrest may elicit apoptosis insomuch that cell death may not only be due to primary damage but also result from accumulation of damages arising from the cell cycle arrest itself [33].

### Oxidative stress in A549 cells

The production of reactive oxygen species (ROS) was evaluated in A549 cells compared with HL60 promyelocytic cells. The HL60 cell line is a cell model that is frequently used to access the oxidant or antioxidant potential of different compounds [34],[35]. In the current study, Selol surprisingly did not induce an oxidative burst on HL60 cells (Figure 7(b)). Otherwise, ROS accumulation was observed in A549 cells at higher SPN exposure times (24, 48 and 72 h) (Figure 7(b)). These findings suggest that the SPN-induced oxidative burst is variable according to the cell type.

Additionally, increased expression of GPX1 (glutathione peroxidase 1) (5-fold and 7-fold at 48 h and 72 h, respectively) was evidenced on A549 cells (Figure 7(a)). Increased levels of ROS coincident with the up-regulation of GPX1 indicate that A549 cells responded in response to exposure to SPN by activation of the antioxidant defense systems. Suchocki et al. (2010) [5] showed the involvement of ROS and the inhibition of CYP1A1 (cytochrome P450) induced by Selol in cervix cancer cell lines. Conversely, in this study, the expression of CYP1A1 (4-fold on both 48 h and 72 h) on A549 cells was enhanced by SPN treatment (Figure 7(a)). The metabolism and detoxification of xenobiotics performed by cytochrome P450 may be associated with SPN metabolism and ROS generation [36]. Therefore, the present study suggests that ROS production on A549 cells may be associated with cell death and inhibition of proliferation, as evidenced by Chen et al. (2013) [37].

Despite the induction of oxidative stress observed on A549 cells, SPN had no effect on the gene expression of catalase at the concentration and times evaluated (Figure 7(b)), corroborating the results of a study on prostate cancer cells treated with Selol for 24 and 48 h [38].

## Conclusions

This study demonstrates that Selol can act as a cytostatic agent and corroborates previous reports indicating that it is effective against cancerous cells and safer for clinical applications than sodium selenite. Furthermore, the present study includes the development of a stable and monodisperse aqueous vehicle for Selol delivery, namely PVM/MA-shelled Selol nanocapsules, which are able to maintain the activity of free Selol against pulmonary adenocarcinoma cells, exhibit reduced toxicity to non-tumor cells *in vitro* and are thus potentially suitable for the treatment of some types of lung cancer. Further *in vivo* studies should be performed to evaluate the potential of this formulation for human therapy.

## Methods

### Materials

Selol composed of 5% selenium (w:w) was provided by Warsaw Medical University (Poland). PVM/MA (Gantrez AN 119) was kindly gifted by ISP Corp. (Brazil). Human lung adenocarcinoma (A549) and promyelocytic leukemia (HL60) cell lines were purchased from American Type Culture Collection (USA). Sodium selenite, trifluoroacetic acid, dimethyl sulfoxide and rhodamine 123 were purchased from Sigma (USA). Dulbecco’s modified Eagle’s medium, F12 medium, Roswell Park Memorial Institute medium, 3,4,5-dimethylthiazol-2,5 biphenyl tetrazolium bromide (MTT), annexin V-FITC and propidium iodide (PI) were provided by Invitrogen (USA). Fetal bovine serum, penicillin and streptomycin were purchased from Gibco (USA). RNase A was obtained from Promega (USA). Primers and 2’ ,7’-dichlorodihydrofluorescein diacetate (DCFH-DA) were acquired from Life Technology (USA). Phorbol 12-myristate 13-acetate (PMA) was obtained from Abcam (England). GeneJet RNA purification and cDNA Maxima kits were provided by Life Science (USA). DNase I, free-RNase kit was purchased from Thermo Scientific (USA). The TaqMan gene expression assay was obtained from AB Applied Biosystems (USA). Grids and supports of copper and osmium tetroxide were obtained from Electron Microscopy Sciences (USA). Dichloromethane was purchased from Vetec (Brazil). Ethanol and acetone were purchased from J. T. Baker (USA). Phosphate buffer saline was obtained from Laborclin (Brazil).

### Preparation of nanocapsules

Nanocapsules were prepared through an interfacial nanoprecipitation method. Briefly, Selol (oil phase) and PVM/MA (surfactant) were dissolved in acetone at room temperature (RT). Next, ethanol and distilled water were sequentially added to the acetone solution, under mild stirring, to form a yellowish, opaque suspension. The organic solvents were removed by distillation at 45°C under reduced pressure (80 mbar) in a rotavapor apparatus (Rotavapor RII®, Buchi Switzerland). Next, the resulting oily nanocapsules were centrifuged at 22,000 × *g* for 30 min, the transparent aqueous supernatant was removed, and the pellet was resuspended in distilled water. This preparation was immediately characterized and/or stored at 4°C until usage.

### Colloidal characterization

The nanocapsules were dispersed in phosphate buffer saline (PBS) (pH 7.4) at a concentration of Selol equivalent to 100 μg/mL. Then, the hydrodynamic diameter (HD) and polydispersity index (PDI) and the zeta potential (ζ potential) were measured at 25°C by dynamic light scattering (DLS) and electrophoretic laser Doppler anemometry (ZetaSizer Nano ZS®, Malvern Instruments), respectively.

### Surface morphology and structure

The shape and surface morphology of the capsules were investigated using a field emission scanning electron microscope (SEM) (JEOL JSM 7001-F®, Japan). Before analysis, the composition was diluted with ultrapure water to 5% (v:v), and 20 μL was deposited onto copper supports. Next, the sample was fixed with 1% osmium tetroxide vapor (w:v) for 1 h, left to dry at RT and coated with gold using a Blazers SCD 050® sputter coater (Blazers Union AG, Liechtenstein). The images were digitized using an UltraScan® camera connected to the Digital Micrograph® 3.6.5 computer software (Gatan, USA).

The diameter values of 300 nanocapsules were measured with the Image Pro-Plus® 5.1 software from images captured with a transmission electron microscope (TEM) (JEOL JEM 1011®, Japan). Before this analysis, the sample was diluted with ultrapure water to 3% (v:v) and deposited onto a copper grid. The dried sample was fixed and contrasted with 1% osmium tetroxide vapor (w:v) for 20 min. The images were digitized using an UltraScan® camera connected to the Digital Micrograph 3.6.5® computer software (Gatan, USA).

### Efficiency of Selol encapsulation

The Selol concentration in the nanocapsules was estimated according to Suchocki et al. (2003) [39]. Briefly, 300 μL of the nanocapsules dispersion was centrifuged at 22,000 × *g* and 4°C for 30 min. The pellet was left to dry at RT for two days. Next, Selol was extracted from the pellet with 800 μL of dichloromethane and oxidized with 200 μL of trifluoroacetic acid. The selenium absorbance was measured at a wavelength of 380 nm in a quartz cuvette. The efficiency of Selol encapsulation was calculated considering the ratio of the encapsulated mass to the mass of Selol that was initially used.

### Thermodynamic stability studies

Storage at room temperature, 4°C and −20°C. Aliquots of the nanocapsule dispersion were stored at RT, 4°C or −20°C, and their colloidal characteristics (HD, PDI and ζ potential) were evaluated every 15 days.

Freezing and thawing cycles. Fifteen cycles of freezing (−20°C) and thawing (25°C) were applied to a nanocapsule aliquot. After each cycle, the HD, PDI and ζ potential were evaluated.

### Cell culture

The A549 human lung adenocarcinoma cell line was cultured with a 1:1 (v:v) mixture of Dulbecco’s modified Eagle’s medium (DMEM) and F12 medium supplemented with 10% (v:v) fetal bovine serum (FBS) and 1% (v:v) antibiotic solution (100 units/mL penicillin and 100 mg/mL streptomycin). Human connective tissue cells harvested from the dental pulp of normal teeth were maintained in primary culture and used as non-tumor control cells, namely normal cells [40]. These cells were grown in DMEM supplemented with 10% FBS and 1% antibiotic solution, as described above. Both cells were maintained at 37°C in a 5% CO_2_ and 80% humidity environment.

### Treatment design

The cells were allowed to adhere to culture microplates for 24 h and were then treated as follows: (1) Selol-PVM/MA nanocapsules (SPN), (2) free Selol (S), (3) blank polymeric nanoparticles without Selol (Bl), and (4) sodium selenite (SS). Cells treated with culture medium, culture medium/acetone and culture medium/PBS corresponded to the control groups of SPN, S and SS, respectively. For the free Selol treatments, Selol was dissolved in acetone and then added to the culture medium, as described by Suchocki et al. (2007) [4]. Before the tests, a viability study was performed to ensure that the volumes of acetone required for each Selol concentration would not be cytotoxic themselves. Bl nanoparticles were prepared using the same method with the same concentrations of the components used for SPN but without Selol. Each treatment was performed in triplicate with different Se concentrations (50, 100 and 150 μg/mL) and times of exposure (24, 48 and 72 h). The concentration of PVM/MA in the treatments with Bl was equivalent to that used in the SPN treatment.

To verify whether the biological activity of SPN was maintained during storage at 4°C, the cell viability was also evaluated on days 1^st^ and 60^th^ after preparation of the SPN.

### Cell viability

The cells were seeded and treated as described above. Next, the cells were incubated with 0.5 mg/mL MTT for 2.5 h. The MTT solution was removed, and formazan was extracted from the cells with dimethyl sulfoxide [41]. The absorbance at a wavelength of 595 nm was measured using a spectrophotometer (Spectramax M2, USA) and was used as an index of cell viability. The results were expressed as percentages relative to the control groups after 24 h of treatment.

### Cell counting

A549 and normal cells were treated with SPN (100 μg/mL) for 24, 48 and 72 h. Next, the cells were harvested and quantified using a Scepter™ Cell Counter (Millipore, USA).

### Morphology and confluence of cells

The morphology and confluence of the cells were analyzed using a phase contrast microscope (Zeiss, Germany) and the AxioVision® software (Zeiss, Germany). For ultrastructural analysis, the cells were fixed, contrasted and dehydrated in agreement with the method described by Carneiro et al. (2011) [42]. Ultrathin sections were observed through TEM, and the images were digitized.

### Real-time cell index

Real-time cell analysis was performed with a RTCA instrument (xCelligence, Roche, Switzerland) [43]. Briefly, A549 cells were seeded for 24 h on plates containing microelectronic sensor arrays and later incubated with medium or treated with different concentrations of SPN (50, 100, and 150 μg/mL). The cells were automatically monitored every 15 min for up to 100 h. Two independent experiments were performed.

### Reactive oxygen species

The intracellular production of reactive oxygen species (ROS) was measured using 2’ ,7’-dichlorodihydrofluorescein diacetate (DCFH-DA) as an oxidation-sensitive fluorescent probe. The ROS production was evaluated in A549 and HL60 cells [34],[35]. The HL60 cells were cultured in RPMI (Roswell Park Memorial Institute medium) supplemented with 10% FBS and 1% antibiotic solution and were maintained at 37°C, 5% CO_2_ and 80% humidity. Then, the cells treated or not treated with SPN (100 μg/mL) for different times (1, 4, 8, 24, 48 and 72 h) were stained with 2.5 μM DCFH-DA for 30 min at 37°C. HL60 cells treated with 10 mM phorbol 12-myristate 13-acetate (PMA) were used as a positive control. A total of 10,000 events per sample were analyzed using a FC500® cytometer (Beckman Coulter, USA) and the FlowJo 7.6.3 software.

### Annexin V-FITC/propidium iodide staining

The externalization of phosphatidylserine and the loss of plasma membrane integrity, which are signs of apoptosis and necrosis, respectively, were assessed with a double-staining kit consisting of FITC-labeled annexin V and propidium iodide (PI). The cells were incubated with 100 μL of binding buffer containing 10 mM HEPES/NaOH (pH 7.4), 140 mM NaCl and 2.5 mM CaCl_2_. Next, 5 μL of annexin V-FITC and 10 μL of PI (50 μg/mL) were added, and the cells were incubated for 15 min in the dark at RT. The cells were analyzed with a CyFlow® space cytometer (Partec, Germany), and 10,000 events were counted per sample. Cells not incubated with Annexin V-FITC and PI were used as the negative control. Cells incubated with 100 μg/mL SS for 24, 48 and 72 h were used as annexin V staining-positive cells. Cells killed by heating at 60°C for 5 min were used as PI staining-positive cells. All of the cytometry results reported in the CyFlow® space were analyzed using the Windows™ Flow Max® and FlowJo 7.6.3 software programs.

### DNA fragmentation and cell cycle analysis

The cell cycle was evaluated by the quantification of total DNA. A549 cells were fixed with 70% ethanol for 2 h at 4°C, rinsed with PBS, incubated with 50 μg/mL RNase A for 30 min at 37°C and stained with 50 μg/mL PI for 30 min at RT [44]. A total of 10,000 events per sample were counted with a CyFlow® cytometer, and the percentage of cells in different phases of the cell cycle was determined. Only those cells presenting DNA content in the range of 2n-4n were considered in the cell cycle analysis. Fragmented DNA was identified in the sub-G1 (DNA content < 2n) population and calculated considering the totality of events.

### Mitochondrial membrane potential

The fluorescent cationic substrate rhodamine 123 (Rho123) was used to assess the mitochondrial membrane potential (*∆Ψm*) in A549 cells. The cells were incubated with 5 μg/mL Rho123 for 15 min at RT and washed twice with PBS [45]. A total of 10,000 events were analyzed per sample using a CyFlow® cytometer.

### Quantitative RT-PCR (qRT-PCR)

A549 cells were harvested after SPN treatment (100 μg/mL) for 24, 48 and 72 h. The total RNA was extracted using a GeneJet RNA purification kit and was then treated with DNase I, free-RNase kit. cDNA was synthesized from mRNA using cDNA Maxima reverse transcription reagents. qRT-PCR was performed using a TaqMan gene expression assay, and the amplification reactions were performed using a Fast Real-time System 7900HT (Applied BioSystems, USA). The following primers (and their specifications) were used: ACTB (Hs99999903_m1), BAX (Hs00180269_m1), BCL2 (Hs00608023_m1), CAT (Hs00156308_m1), CDC25C (Hs00156411_m1), CCNB1 (Hs01030099_m1), CCND1 (Hs00765553_m1), CCNE1 (Hs01026536_m1), CYP1A1 (Hs00153120_m1), GAPDH (Hs02758991_g1), GPX1 (Hs00829989_gH), SIRT3 (Hs00953477_m1), and WEE1 (Hs00268721_m1). All of the kits were used according to the manufacturer’s instructions.

The cDNA dilutions were defined based on threshold cycles (CT) (17 – 20) derived from the amplification of the constitutive gene GAPDH (R^2^ ≥ 0.9974). Each sample was normalized based on the mRNA expression level of GAPDH. The gene expression values were obtained using the 2^-∆∆CT^ equation. A gene was considered to be differentially expressed when the transcript rate (FC, fold change) was at changed by at least twofold compared with the untreated control sample [46].

### Statistical analysis

All of the experiments were performed in triplicate and repeated three times. The results are represented as the means ± standard deviation. Significant differences were assessed by one- or two-way analyses of variance followed by Tukey or Bonferroni’s post-tests (*α* = 0.05) using the GraphPad Prism 5.0 software.

## Competing interests

We confirm that we have given due consideration to the protection of intellectual property associated with this work and that there are no impediments to publication, including the timing of publication, with respect to intellectual property. The authors disclose no potential conflicts of interest.

## Authors’ contributions

LRS was the principal investigator and took primary responsibility for the paper. LRS, LAM, RBA and SNB participated in the design and coordination of the study. EM and PS conducted the Selol development. LAM, RG and PCM developed and characterized the Selol nanocapsules. LRS, MSCS, RS and GAJ performed the biological assays. AG participated in the coordination of some experiments and helped draft the manuscript. LRS, LAM, RBA and SNB wrote the manuscript and all of the authors helped discuss the results, adding thoughtful insights to the manuscript, and approved the final manuscript.
